# Subchorionic hematoma: Research status and pathogenesis (Review)

**DOI:** 10.3892/mi.2024.134

**Published:** 2024-01-19

**Authors:** Tiantian Xu, Weiwei Lun, Yuanfang He

**Affiliations:** Department of Obstetrics and Gynecology, The Third Affiliated Hospital of Zhengzhou University, Zhengzhou, Henan 450052, P.R. China

**Keywords:** subchorionic hematoma, pathogenesis, etiology, treatment

## Abstract

Subchorionic hematoma (SCH) is a hematoma in which blood accumulates between the chorion and decidua basalis due to the separation of the chorion and decidua basalis. It is common in patients with threatened abortion in early pregnancy and is mainly detected by ultrasound. SCH mainly manifests as an hypoechoic or anechoic crescent-shaped fluid dark area on ultrasound images. Although there are numerous studies on SCH, its pathogenesis and etiology remain unclear, and its influence on pregnancy outcomes is also controversial; there are also no uniform clinical treatment guidelines. Current studies suggest that the occurrence of SCH may be related to several factors, such as abnormal coagulation function, autoimmune factors of pregnant women, assisted reproduction, drug use during pregnancy and reproductive tract infection; however, its exact etiology remains unclear. Some studies suggest that SCH is associated with adverse pregnancy outcomes such as miscarriage, preterm birth, preeclampsia and fetal growth restriction, although other studies have found that SCH does not increase the risk of adverse pregnancy outcomes. Therefore, the present review mainly discusses the pathogenesis, etiology and treatment of SCH in an aim to provide a reference for the clinical treatment of this condition in pregnant women.

## 1. Introduction

A subchorionic hematoma (SCH) refers to the separation and bleeding of the chorion and decidua, resulting in the accumulation of blood between them. It mainly manifests as a crescent-shaped, triangular, or irregular hypoechoic or hyperechoic liquid dark area on ultrasound images (1). The clinical incidence rate of SCH is 0.48-39.5% (2). To date, a few studies have been conducted on the etiology and pathogenesis of SCH, mainly focusing on the following aspects: Coagulation dysfunction, immune factors, reproductive tract infections, assisted reproductive technology and drugs (3-5). Although there is ample experience in the clinical treatment of this condition, a unified treatment method does not yet exist. Therefore, the present review mainly discusses the pathogenesis, etiology and treatment of SCH in an aim to provide a reference for the treatment of the disease and reduce the occurrence of adverse pregnancy outcomes.

## 2. Pathogenesis of SCH

Placental formation during early pregnancy is a critical step in fetal development. The impairment of trophoblast invasion and angiogenic capacity during early placental formation can lead to increased vascular fragility. When the placenta bleeds, blood is removed along the path of least resistance and may accumulate between the chorion and decidual tissue outside, resulting in the formation of SCH. It has been suggested that some factors cause syncytiotrophoblast cells in the outer chorionic layer to invade and expand into the decidua during early pregnancy, accompanied by impaired angiogenesis of the decidua, leading to separation and bleeding between the decidua and chorion, as well as in the formation of hematoma and fetal membrane peeling (6).

## 3. Etiology of SCH

### Abnormal coagulation function

The hypercoagulable state of maternal blood is a high-risk factor for adverse pregnancy outcomes. Recently, it has been proposed that SCH formation is closely related to the hypercoagulable state of maternal blood (7). Thrombosis may be caused by the contraction of blood vessels, platelet aggregation and damage to the decidual vascular endothelium, leading to the occlusion of veins in the subchorionic space, which prevents blood from flowing out (8). In addition, subchorionic blood flow forms a vortex and subchorionic fibrin aggregates, which leads to blood accumulation in the subchorionic space to form a hematoma. As a coagulation protein, fibrinogen can promote high levels of platelet aggregation, cause vascular endothelial damage, and increase the risk of thrombosis (9). Therefore, an increase in the levels of fibrinogen in pregnant women may increase the risk of hematoma formation (10). In a previous study, Hu *et al* (11) examined 30 patients with SCH and observed that the proportion of pregnant women with an acquired prethrombotic state during pregnancy was as high as 70%; this also suggested the importance of screening maternal coagulation function in early pregnancy.

### Immune dysfunction

According to the allogeneic transplantation theory, maternal immune tolerance is the basis of fetal survival in mothers. Maternal immune abnormalities, such as autoantibodies, may increase the risk of SCH. Anticardiolipin antibodies are the signature antibodies of antiphospholipid syndrome, which can induce platelet aggregation, lead to thrombosis and are associated with recurrent miscarriage. Antinuclear antibodies can also lead to hyperimmunity and induce complement activation, leading to embryo arrest (12). Li *et al* (13) observed that the positivity rate of autoantibodies in patients with SCH was significantly higher than that in normal pregnant women, and high titers of antinuclear antibodies were more likely to be a high-risk factor for SCH. Recent studies have demonstrated that an imbalance in Th1/Th2 immune cells plays a crucial role in the pathogenesis of SCH. Cytokines produced by Th1 cells mainly mediate cellular immunity and inflammatory responses that can inhibit embryo implantation, trophoblast growth, embryonic development and fetal survival (14,15). Cytokines secreted by Th2 cells mainly mediate humoral immunity, which maintains pregnancy and antagonizes Th1 type cytokines. Therefore, Th1 dominance causes immune damage during pregnancy, whereas Th2 dominance is beneficial for the maintenance of pregnancy. In patients with SCH, an imbalance between the two cytokines is observed, with a shift to Th1-type cytokines (16). The aforementioned studies suggest that the formation of SCH may be related to disorders of the maternal immune system; however, the specific underlying mechanisms remain unclear; therefore, further research is required.

### Assisted reproductive technology

It has been found that the incidence of SCH in pregnant women with assisted reproduction is higher than that in women with natural pregnancy, and frozen embryo transfer and blastocyst transfer increase the risk of SCH (17). A previous study demonstrated that the transfer of poor blastocyst trophoblast cells can notably increase the risk of SCH, which may be related to the long-term *in vitro* culture of blastocysts and abnormal placental development (18). So *et al* (19) assumed that uterine cavity surgeries, such as endometrial polyp removal and hysteroscopy prior to embryo transfer, would damage the endometrium and affect the normal development of the placenta. Individuals receiving assisted reproductive technology may also have immune diseases, coagulation abnormalities and other diseases that may induce SCH. The increased occurrence of SCH caused by assisted reproductive technology may be related to the increased estradiol level in patients; however, some researchers observed that during the process of embryo transfer, high serum estradiol levels were not associated with an increased risk of SCH formation (20).

## 4. Treatment of SCH

Although there is ample experience in the clinical treatment of SCH, clear guidelines are not yet available for the treatment of this condition. Clinically, the majority of patients receive symptomatic treatments, such as uterine contraction inhibition, hemostasis and infection prevention. Previous studies have demonstrated that immunomodulatory drugs may play a role in the treatment of patients with immune disorders (21-23).

### Immunoglobulin

Immunoglobulin is an immunomodulator, which is typically used to treat certain autoimmune diseases. Immunoglobulin contains an idiotypic antibody against the placental trophoblast antigen. Therefore, it can be used in patients with recurrent spontaneous abortion who have insufficient autoantibody production. It has been suggested that the mechanism of immunotherapy may include reducing the level and toxicity of natural killer (NK) cells and regulating the production of Th1 and Th2 cytokines (24,25). The study by Tao *et al* (26) demonstrated that immunoglobulins could notably reduce the number of CD56^+^ NK cells in the peripheral blood of patients, thereby improving patient outcomes. Ahmadi *et al* (23) demonstrated that the number and toxicity of NK cells in the peripheral hematomas of patients treated with immunoglobulins markedly decreased in the third trimester, the level of inhibitory receptors of NK cells increased and the level of activated receptors notably decreased. Another study demonstrated that the use of immunoglobulins in patients with recurrent spontaneous abortion can reduce the production of Th1 cytokines and induce the release of Th2 cytokines from peripheral blood immune cells, thereby maintaining the balance between Th1 and Th2 cytokines and improving pregnancy outcomes (27).

### Alpha-lipoic acid (ALA)

ALA, a natural antioxidant with anti-inflammatory properties, regulates the secretion of inflammatory factors and blocks the activation of NK cells. ALA has selective immunomodulatory activity, which can regulate the secretion of inflammatory cytokines, increase the number of Tregs, inhibit the production of vascular and cell adhesion molecules, reduce the expression of CD4 on blood single and cell surfaces, block NK cell activation and reduce cytotoxicity (28,29). The drug can be administered either vaginally or orally. A randomized controlled trial tested the simultaneous vaginal administration of lipoic acid and progesterone in patients with SCH and threatened abortion, and patients administered lipoic acid were observed to have more rapid hematoma absorption; hence, ALA was considered for use in the treatment of patients with SCH (30). Concurrently, some studies have observed that the therapeutic effects of oral lipoic acid and vaginal progesterone are more significant than those of vaginal progesterone alone (30,31); hence, α-lipoic acid is considered to be a potential drug for the treatment of SCH. In the future, further prospective experiments with larger sample sizes are required to further explore the therapeutic mechanisms.

### Other therapeutic drugs

Traditional Chinese medicine (TCM) has recently become a popular topic in clinical research for the treatment of SCH. An increasing number of studies have indicated that TCM for tonifying kidney and promoting blood circulation has obvious effects on the absorption and improvement of the clinical symptoms of SCH (32-34). TCM assumes that SCH is caused by spleen and kidney ‘qi’ deficiency and blood stasis, while it includes therapies, such as tonifying the kidneys and spleen, promoting blood circulation, and removing blood stasis to promote the absorption of SCH and improve the symptoms of abortion (35). A previous study demonstrated that the Shoutai Pill combined with progesterone for the treatment of threatened abortion with SCH promoted hematoma absorption and improved abdominal pain symptoms (36). Subsequently, another study demonstrated the potent effect of Bushen Yiqi decoction in the treatment of SCH, which can regulate the balance of Th1/Th2 cytokines and downregulate the level of NK cells in peripheral blood, thus improving immune regulation disorders in patients with SCH (37).

A close association has been observed between early maternal serum progesterone and HCG levels and pregnancy outcomes of pregnant women. Therefore, progesterone and dydrogesterone are commonly used in the treatment of patients with SCH and threatened abortion. Both are progesterone-rich drugs that promote endometrial proliferation and improve the excitability of endometrial smooth muscle cells, while reducing uterine contraction. Therefore, they can be used to treat patients with abortions (38). In addition, dydrogesterone can induce the production of progesterone-inducible blocking factor (PIBF) by lymphocytes, inhibit the activity of NK cell continuation and can maintain pregnancy. A previous study revealed that the use of progesterone combined with dydrogesterone improved the shift in Th1/Th2 cytokines in patients with SCH, thereby improving the immune imbalance and reducing the occurrence of adverse pregnancy outcomes (39). Additionally, progesterone can reduce the cytotoxicity of decidual NK cells by blocking the expression of perforin and degranulation of decidual NK cells by PIBF (40).

## 5. Conclusion and future perspectives

In summary, SCH is associated with coagulation dysfunction, immune dysfunction and assisted reproductive technology. Although clinical research is increasingly abundant, basic research is limited and mainly focuses on the adverse effects of SCH on pregnancy outcomes. Therefore, more basic experiments are required to further investigate these pathological mechanisms. In-depth analysis of the causes of hematoma, the study of the association between hematoma and pregnancy outcomes, and the exploration of potential treatment methods may help to provide a more in-depth understanding of SCH. This may also provide appropriate treatment strategies for patients with SCH in early pregnancy and may reduce the psychological pressure on pregnant women and the occurrence of adverse pregnancy outcomes.

## Data Availability

Not applicable.
